# The Two-Year Outcomes of Phacoemulsification Combined with GATT Versus Standalone GATT in Open-Angle Glaucoma: A Comparative Study

**DOI:** 10.3390/diagnostics15050542

**Published:** 2025-02-24

**Authors:** Zübeyir Yozgat, Mehmet Cem Sabaner

**Affiliations:** Department of Ophthalmology, Kastamonu University, Kastamonu 37150, Turkey; drmcemsabaner@yahoo.com

**Keywords:** glaucoma surgery, minimally invasive glaucoma surgery, gonioscopy-assisted transluminal trabeculotomy, phacoemulsification, combined surgery

## Abstract

**Background/Objectives**: The aim of this paper was to evaluate the two-year outcomes of phacoemulsification combined with gonioscopy-assisted transluminal trabeculotomy (PHACO-GATT) versus standalone GATT in terms of efficacy, safety, and surgical success. **Methods**: This retrospective, comparative study included 64 eyes of 64 patients with moderate-to-severe open-angle glaucoma: 35 patients (54.7%) with primary open-angle glaucoma (POAG) and 29 patients (45.3%) with pseudoexfoliative glaucoma (PEG). Group 1 (*n* = 38) underwent PHACO-GATT, and Group 2 (*n* = 26) underwent standalone GATT. Data, including intraocular pressure (IOP), the number of anti-glaucomatous medications, and complications, were analyzed preoperatively and postoperatively (1st, 3rd, 6th, 12th, and 24th months). Surgical success was defined as achieving a ≥ 20% IOP reduction or IOP ≤ 21 mmHg with or without medications. **Results**: The mean age was 74.4 ± 7.2 years in Group 1 and 70.8 ± 7.3 years in Group 2. Both groups achieved significant IOP reductions at the 24-month follow-up: Group 1 from 28.6 ± 6.3 mmHg to 12.7 ± 2.4 mmHg, and Group 2 from 27 ± 4.8 mmHg to 13 ± 1.7 mmHg (both *p* < 0.001). BCVA in Group 1 improved significantly from 0.77 ± 0.29 logMAR to 0.28 ± 0.13 logMAR at 24 months (*p* < 0.001), while in Group 2, it remained stable at 0.46 ± 0.19 logMAR at baseline and 0.47 ± 0.19 logMAR at 24 months (*p* > 0.05). The mean number of anti-glaucoma medications decreased significantly in both groups (*p* < 0.001) without significant intergroup differences (*p* > 0.05). Complication-free rates were 68.4% in Group 1 and 69.2% in Group 2 (*p* = 0.899). Surgical success rates were comparable between groups at 12 (100% in both groups) and 24 months (94.7% in Group 1, 96.2% in Group 2). **Conclusions**: Both PHACO-GATT and standalone GATT demonstrated comparable efficacy and safety over a two-year period. PHACO-GATT provided significant visual acuity improvements due to cataract extraction, making it a suitable option for patients with coexisting cataracts and glaucoma.

## 1. Introduction

Glaucoma and cataracts are among the leading causes of visual impairment globally, particularly in aging populations, where their prevalence continues to rise [1,2]. Managing these coexisting conditions often requires a surgical intervention, and combined procedures have gained traction for their ability to minimize surgical trauma, reduce healthcare costs, and eliminate the need for sequential surgeries [3].

Traditional trabeculectomy has long been the gold standard for surgical glaucoma management due to its effectiveness in lowering intraocular pressure (IOP) [4]. However, significant risks such as hypotony, bleb-related complications, and infections have prompted the development of minimally invasive glaucoma surgeries (MIGSs). MIGS techniques aim to achieve effective IOP reduction while offering fewer complications and faster recovery times compared to trabeculectomy. Among these, gonioscopy-assisted transluminal trabeculotomy (GATT), first described by Grover et al., has emerged as a promising ab interno approach [5]. By targeting the trabecular meshwork (TM) and Schlemm’s canal, GATT aims to restore the physiological outflow of aqueous humor without requiring conjunctival or scleral incisions; however, its efficacy may vary among patients. This bleb-free and sutureless procedure has demonstrated a comparable IOP reduction to trabeculectomy, with a lower risk of complications [5,6]. The GATT procedure can help reduce postoperative complications; however, it does not guarantee 100% efficacy. Additionally, it preserves the conjunctiva and sclera, potentially facilitating future filtration surgeries if needed.

Phacoemulsification (PHACO) surgery has been shown to have a long-term IOP-lowering effect in both glaucomatous and non-glaucomatous eyes [7]. This effect can be explained by several mechanisms, including an increase in angle width when replacing a 4–5 mm cataractous lens with a 1 mm intraocular lens, traction on the ciliary body via the zonules, changes in TM related to induced inflammation, and an increase in prostaglandin F2 [7,8]. Combined MIGS and cataract surgery, such as phacoemulsification with GATT, provides a comprehensive approach to managing glaucoma and cataracts simultaneously. Studies, including Grover et al.’s study of 2-year outcomes in 198 patients with open-angle glaucoma, have shown that PHACO-GATT achieves significant IOP reduction and improved visual acuity, alongside decreased dependency on anti-glaucoma medications [6,9,10,11,12,13,14]. Moreover, evidence highlights that PHACO-GATT offers a safe and effective option for patients with coexisting cataracts and glaucoma [9,10,11,12,13,14].

Despite the growing adoption of combined MIGS and cataract surgery, there is limited data directly comparing the long-term outcomes of PHACO-GATT and standalone GATT. This study aims to address this gap by evaluating and comparing the two-year outcomes of these surgical approaches in terms of efficacy, safety, and surgical success. The findings will provide valuable insights for optimizing treatment strategies in patients with moderate-to-severe open-angle glaucoma.

## 2. Materials and Methods

### 2.1. Study Design

This retrospective comparative study included 64 eyes of 64 patients with moderate-to-severe open-angle glaucoma, of which 35 patients (54.7%) were diagnosed with primary open-angle glaucoma (POAG) and 29 patients (45.3%) with pseudoexfoliative glaucoma (PEG), who underwent either PHACO combined with GATT (Group 1, *n* = 38) or standalone GATT surgery (Group 2, *n* = 26). Data, including demographic characteristics, IOP, the number of anti-glaucoma (AG) medications, complications, and surgical success rates, were collected from patient records and analyzed. The surgical procedure, postoperative care, and follow-up were standardized for all patients to ensure consistency.

All procedures were performed by following the tenets of the Declaration of Helsinki. Institutional review board approval was obtained from the local ethics committee (IRB:2024-KAEK-139/10 December 2024). Informed consent was obtained from all patients.

### 2.2. Inclusion and Exclusion Criteria

The inclusion criteria were as follows: (1) patients with moderate-to-severe open-angle glaucoma whose IOP could not be controlled despite maximum medical treatment; (2) those with vision-threatening cataracts (best-corrected visual acuity above 0.3 (logMAR)); (3) patients undergoing 360-degree suture trabeculotomy; (4) adult patients ≥ 18 years old; and (5) intolerance to medical treatment.

The main exclusion criteria were as follows: (1) those with a history of any previous ocular surgery other than PHACO; (2) patients with glaucoma rather than open-angle glaucoma; (3) those with corneal pathology that prevents the visualization of TM or measurement of IOP; and (4) corneal and/or lens opacities that may affect OCT imaging.

### 2.3. Surgical Procedures

All procedures were performed by two experienced consultant glaucoma surgeons (EY and ZY). All patients had at least 2-year follow-up data. All surgeries were performed under local anesthesia. Following the use of topical 0.5% propacaine preoperatively, standard sterile surgical preparation was performed. Phacoemulsification surgery was started by opening side ports from the upper and lower corneal quadrants with a 20-gauge MVR blade. Intracamerally, preservative-free 1% lidocaine was used for anesthesia. Then, a dispersive viscoelastic (Sodium hyluronate, Protectalon 3%, VSY Biotechnology, Istanbul, Turkey) was injected into the anterior chamber. A tunnel incision was made through the clear cornea adjacent to the limbus with a 2.4 mm keratome knife in the temporal quadrant. A round capsulorhexis with a diameter of 5 mm was made by entering through the clear corneal incision with capsulorhexis forceps. Hydrodissection was performed by administering a balanced salt solution under the capsule with a hydrodissection needle, and the nucleus was rotated. By entering the anterior chamber with the PHACO handpiece (Signature Pro, Johnson and Johnson Vision, North Jacksonville, FL), the nucleus was first divided into two with the Direct Chop method, and then the half pieces were divided, emulsified, and cleaned by a PHACO type and vacuum. Cortical residues were cleaned by irrigation and aspiration cannulas and bimanual irrigation and aspiration. A viscoelastic was administered to the anterior chamber, and a foldable monoblock hydrophobic intraocular lens (Sensar, Johnson & Johnson Surgical Vision; Santa Ana, CA, USA) was implanted into the bag. An anterior chamber was created by hydrating the side ports with balanced salt solution.

For the GATT surgery, the operating microscope was tilted 30° toward the surgeon, while the patient’s head was tilted 30° away from the surgeon, according to the gonioscopy surgical protocol. Side ports were opened from the corneal superonasal and inferonasal quadrants with a 20-gauge MVR blade. The anterior chamber was filled with a dispersive viscoelastic (Sodium hyaluronate, Protectalon 3%, VSY Biotechnology, Istanbul, Turkey). The Swan-Jacob gonioprism lens (Ocular Instruments, Bellevue, WA, USA) was placed to view the SC in the nasal quadrant. By entering through the temporal incision made for PHACO surgery, an approximately 1–1.5 mm incision (goniotomy) was created at the angle with the MVR knife. The 5/0 prolene suture placed in the anterior chamber through the inferonasal/superonasal incision was grasped and placed into the goniotomy with microforceps placed in the anterior chamber through the temporal incision. The 5/0 prolene suture was pushed forward 360 degrees circumferentially through Schlemm’s canal. Then, a 360-degree trabeculotomy was performed by holding the distal edge of the suture coming out of the goniotomy and pulling the proximal edge out of the temporal incision (traction). The intraocular viscoelastic was cleaned by irrigation/aspiration. At the end of the operation, 0.1 cc of air and 0.1 cc of cohesive viscoelastic were injected into the anterior chamber (AC) to decrease hemorrhage reflux from the episcleral veins. The side ports were rapidly hydrated to prevent hypotonia.

### 2.4. Postoperative Care and Follow-Up

Patients were evaluated preoperatively and at the following postoperative intervals: day 1, week 1, and months 1, 3, 6, 12, and 24. Assessments included BCVA (best-corrected visual acuity), IOP (measured by Goldmann applanation tonometry), RNFL (retinal nerve fiber layer thickness, via OCT) (Cirrus HD-OCT 5000, Carl Zeiss Meditec AG, Inc., Dublin, CA, USA), and documentation of postoperative complications. Standard postoperative regimens of topical antibiotics and anti-inflammatory medications were applied.

Complete success was defined as achieving an IOP reduction of ≥20% or ≤21 mmHg without AG medications. Qualified success was achieving the same with a maximum of two medications. Surgical failure was defined as requiring additional IOP-lowering surgery.

### 2.5. Statistical Analysis

Data were analyzed using SPSS version 25.0. Continuous variables were summarized as mean ± standard deviation (SD) or median (interquartile range [IQR]), and categorical variables as frequencies and percentages. Between-group comparisons utilized *t*-tests or Mann–Whitney U tests for continuous data, and chi-square or Fisher’s exact tests for categorical data. Longitudinal data, such as IOP and medication changes, were analyzed with repeated-measures ANOVA or Friedman tests, with post hoc pairwise comparisons performed using paired *t*-tests or Wilcoxon signed-rank tests, applying Bonferroni correction for multiple comparisons. Statistical significance was set at *p* < 0.05.

## 3. Results

### 3.1. Baseline Characteristics

This study included 64 eyes of 64 patients, divided into Group 1 (PHACO-GATT, *n* = 38) and Group 2 (standalone GATT, *n* = 26). Baseline demographic and ocular characteristics are summarized in Table 1. The mean age was 74.4 ± 7.2 years in Group 1 and 70.8 ± 7.3 years in Group 2, with no significant difference (*p* = 0.061). The gender distribution was also similar between the groups, with 39.5% females in Group 1 and 38.5% females in Group 2 (*p* = 0.935) (Table 1).

Baseline BCVA values differed significantly between the groups, with Group 1 showing worse BCVA (0.77 ± 0.29) compared to Group 2 (0.46 ± 0.19) (*p* < 0.001). This reflects the inclusion of cataract cases in Group 1, which contributed to their reduced visual acuity at baseline (Table 1).

Among all patients, 35 (54.7%) had POAG and 29 (45.3%) had PEG, with no significant differences between the groups (*p* = 0.911). The baseline IOP was 28.6 ± 6.3 mmHg in Group 1 and 27 ± 4.8 mmHg in Group 2 (*p* = 0.365). Similarly, the mean number of AG medications at baseline was 3.4 ± 0.8 in Group 1 and 3.7 ± 0.5 in Group 2 (*p* = 0.182).

No statistically significant differences were found in other baseline ocular parameters, including the cup-to-disc ratio, RNFL thickness, and central corneal thickness (all *p* > 0.05).

### 3.2. Intraocular Pressure Changes

Both groups showed significant IOP reductions from baseline to all postoperative time points (*p* < 0.001, Table 2). At the 24-month follow-up, the mean IOP in Group 1 decreased from 28.6 ± 6.3 mmHg to 12.7 ± 2.4 mmHg, and in Group 2 from 27 ± 4.8 mmHg to 13 ± 1.7 mmHg. Intergroup comparisons revealed no significant differences in IOP reductions at any follow-up visit, including the final visit at 24 months (*p* = 0.259, Table 3).

### 3.3. Anti-Glaucomatous Medication Use

The number of anti-glaucoma medications decreased significantly in both groups postoperatively (*p* < 0.001, Table 4). At baseline, the mean number of medications was 3.4 ± 0.8 in Group 1 and 3.7 ± 0.5 in Group 2. By 24 months, medication use had decreased to 1.2 ± 1 in Group 1 and 1.1 ± 0.8 in Group 2. There were no statistically significant differences in medication reductions between the groups at any follow-up visit (*p* > 0.05).

### 3.4. Complications and Surgical Success

The most frequently observed complication was transient microhyphema, occurring in 26.3% of cases in Group 1 and 23.1% in Group 2. Macrohyphema was observed in 2.6% of cases in Group 1 and 3.8% in Group 2. Both microhyphema and macrohyphema resolved spontaneously without the need for additional surgical intervention. Additionally, transient IOP spikes were recorded in 2.6% of Group 1 and 3.8% of Group 2. All complications were managed conservatively. There were no cases of vision-threatening complications, hypotony, or bleb-related infections in either group.

The rates of complication-free outcomes were similar between the two groups (68.4% in Group 1 vs. 69.2% in Group 2, *p* = 0.899, Table 5). Complete surgical success, defined as achieving an IOP reduction of ≥20% or ≤21 mmHg without medications, was achieved in 31.6% of Group 1 and 26.9% of Group 2 at 24 months (*p* = 0.876). Qualified success, defined as the same IOP criteria with up to two medications, was achieved in 94.7% of Group 1 and 96.2% of Group 2 at 24 months (*p* = 0.867). The surgical failure rate was low, with 5.3% in Group 1 and 3.8% in Group 2 requiring additional IOP-lowering surgery (*p* > 0.05).

### 3.5. Visual and Structural Outcomes

Visual acuity improved significantly in Group 1 postoperatively, while no significant improvement was observed in Group 2. In Group 1, BCVA improved from 0.77 ± 0.29 at baseline to 0.24 ± 0.12 at 1 year and 0.28 ± 0.13 at 2 years, with a statistically significant difference between visits (*p* < 0.001). In contrast, Group 2 demonstrated stable BCVA values, with 0.46 ± 0.19 at baseline, 0.47 ± 0.2 at 1 year, and 0.47 ± 0.19 at 2 years, showing no significant differences between visits (baseline vs. 1 year, *p* = 0.256; baseline vs. 2 years, *p* = 0.119; 1 year vs. 2 years, *p* = 0.680).

When BCVA was compared between the groups, Group 1 showed significantly better visual outcomes at all follow-up visits (*p* < 0.05). The significant improvement in Group 1 can be attributed to the cataract surgery performed as part of the combined procedure, which provided additional visual benefits. In contrast, Group 2 did not undergo cataract surgery, leading to stable but unimproved visual acuity over time.

Retinal nerve fiber layer (RNFL) thickness showed no significant intergroup differences at any time point (*p* > 0.05 for all comparisons). In Group 1, the mean RNFL thickness was 67.8 ± 15.1 µm at baseline, decreasing slightly to 65.9 ± 14.8 µm at 24 months. In Group 2, the mean RNFL thickness was 73.6 ± 11.2 µm at baseline, decreasing to 70.2 ± 13.2 µm at 24 months. The between-group *p* values for RNFL thickness ranged from 0.053 to 0.157 across all time points, indicating no statistically significant differences.

## 4. Discussion

This study compared the two-year outcomes of combined PHACO-GATT and standalone GATT in patients with moderate-to-severe OAG. Both procedures demonstrated significant and comparable reductions in IOP and medication use, with favorable surgical success rates and a low complication profile. These findings contribute to the growing body of evidence supporting MIGS as an effective alternative to traditional filtration surgeries. The dual approach of PHACO-GATT, addressing both glaucoma and cataract simultaneously, offers particular advantages in older populations where these conditions often coexist. In this study, the mean age of patients undergoing PHACO-GATT was 74.4 ± 7.2 years, and the procedure demonstrated significant IOP reduction and medication-sparing effects, alongside visual acuity improvements. These findings highlight PHACO-GATT as an effective strategy for optimizing outcomes in elderly patients with coexisting POAG and cataract [5,15].

GATT, as a blebless and conjunctival-sparing procedure, avoids many of the severe complications associated with traditional trabeculectomy, such as hypotony, bleb leaks, or infections [16,17]. Furthermore, by preserving the conjunctiva, GATT maintains the possibility of future filtering surgeries if required. PHACO-GATT extends these benefits by simultaneously addressing the refractive needs of patients, as cataract extraction not only improves visual acuity but also contributes to a modest IOP reduction through mechanisms such as increased anterior chamber depth and improved trabecular outflow [7,8,14,18].

The significant IOP reductions observed in both the PHACO-GATT and standalone GATT groups in this study align with previously reported outcomes in the literature. At 24 months, the mean IOP reduction was 55.6% in the PHACO-GATT group and 51.9% in the standalone GATT group, with no statistically significant difference between the groups. These results are consistent with the findings of Wan et al., who reported a 39% reduction in IOP at 24 months in a cohort undergoing combined PHACO-GATT, and Sharkawi et al., who demonstrated a 52% reduction in IOP in patients with pseudoexfoliative glaucoma treated with GATT alone [14,15].

The observed reductions in IOP in this study also reflect the efficacy of GATT in addressing the primary pathophysiology of OAG by improving aqueous outflow through a circumferential incision in the trabecular meshwork. This mechanism bypasses resistance within the Schlemm’s canal, allowing for significant pressure reduction without the formation of a bleb [5,16]. Furthermore, the comparable results between PHACO-GATT and GATT alone highlight the efficacy of GATT as a standalone procedure, further emphasizing the effectiveness of IOP in patients with pseudophakia.

The reduction in anti-glaucoma medication use observed in both the PHACO-GATT and standalone GATT groups highlights the efficacy of these procedures in minimizing dependence on pharmacological therapy. In this study, the mean number of medications decreased from 3.4 ± 0.8 to 1.2 ± 1.0 in the PHACO-GATT group and from 3.7 ± 0.5 to 1.1 ± 0.8 in the standalone GATT group at 24 months. This significant reduction is consistent with the findings of Al Habash et al., who reported a decrease from 3.4 to 1.1 medications following PHACO-GATT combined with ab interno canaloplasty procedures, and Sharkawi et al., who demonstrated a reduction to 1.0 medications in patients with PEG treated with standalone GATT [9,15].

This reduction in medication use not only improves patient adherence but also reduces the burden of medication-related side effects, which are often a significant concern in older populations with OAG. The comparable outcomes in medication sparing between the two groups in this study further validate the effectiveness of GATT in restoring physiological outflow and achieving long-term pressure control, irrespective of whether it is combined with cataract surgery. These results reinforce the role of MIGS procedures, such as GATT, in optimizing glaucoma management while reducing the need for intensive medical therapy.

The improved visual outcomes observed in this study highlight the added benefit of cataract extraction in combined PHACO-GATT procedures. In our cohort, BCVA significantly improved from 0.77 ± 0.29 logMAR at baseline to 0.28 ± 0.13 logMAR at 24 months in the PHACO-GATT group (*p* < 0.001). This significant improvement is consistent with the findings of Wan et al., who reported a mean improvement from 0.75 ± 0.43 logMAR to 0.22 ± 0.18 logMAR over 24 months following PHACO-GATT, showing similar trends in visual recovery [14]. In contrast, BCVA in the standalone GATT group remained stable, with values of 0.46 ± 0.19 at baseline, 0.47 ± 0.2 at 1 year, and 0.47 ± 0.19 at 2 years (*p* > 0.05 across visits). Similarly, Sharkawi et al. found no significant changes in BCVA in their standalone GATT cohort, with a mean of 0.3 ± 0.3 logMAR before surgery, 0.2 ± 0.4 logMAR at 12 months (*p* = 0.155), and 0.4 ± 0.8 logMAR at 24 months (*p* = 0.608) [15]. These findings reinforce that standalone GATT does not result in significant visual improvement, likely due to the absence of cataract extraction. In contrast, the dual benefits of PHACO-GATT highlight its advantage in improving visual acuity while also enhancing glaucoma management by increasing anterior chamber depth and facilitating aqueous outflow. This combined approach reduces the need for separate surgical interventions, minimizing patient burden and cumulative surgical risk. These results emphasize the value of combined MIGS and cataract procedures in providing comprehensive care for patients with coexisting cataracts and glaucoma, particularly in older populations where these conditions frequently coexist.

The structural stability observed in RNFL thickness further supports the efficacy of both PHACO-GATT and standalone GATT in controlling glaucoma progression. In our study, RNFL thickness in the PHACO-GATT group showed a slight decrease from 67.8 ± 15.1 µm at baseline to 65.9 ± 14.8 µm at 24 months, while in the standalone GATT group, it decreased from 73.6 ± 11.2 µm to 70.2 ± 13.2 µm. These changes were not statistically significant between the groups (*p* > 0.05 at all time points). This aligns with the findings of Hamze et al., who demonstrated that RNFL thinning over time was minimal and not significantly different in patients undergoing GATT combined with phacoemulsification, and Baykara et al., who reported stable structural outcomes following standalone GATT [16,18]. The preservation of RNFL thickness in both groups indicates the effective control of glaucoma progression, as RNFL thinning is a hallmark of ongoing optic nerve damage. By improving aqueous outflow and reducing IOP, GATT reduces stress on the optic nerve head, which may help preserve RNFL thickness over time. Furthermore, the lack of significant differences in structural outcomes between the two groups underscores the efficacy of GATT as a standalone procedure, even in the absence of cataract surgery. These findings suggest that both PHACO-GATT and standalone GATT are effective in preventing glaucomatous damage to the optic nerve, with minimal structural compromise over two years. This reinforces the role of GATT as a safe and reliable procedure for glaucoma management, particularly in patients with moderate-to-severe disease.

The complication rates and surgical success observed in this study further demonstrate the safety and efficacy of both PHACO-GATT and standalone GATT. In our study, the overall rate of complication-free outcomes was 68.4% in the PHACO-GATT group and 69.2% in the standalone GATT group, with no statistically significant difference between the two groups (*p* = 0.899). These findings align with previous studies reporting low complication rates for both procedures. For instance, Wan et al. reported a similarly low incidence of vision-threatening complications over 24 months in patients undergoing PHACO-GATT, while Sharkawi et al. noted a 2.9% complication rate in patients with pseudoexfoliative glaucoma treated with standalone GATT [14,15]. Hyphema was the most commonly observed complication in both groups in this study, resolving spontaneously in all cases without further intervention. This is consistent with the findings of Baykara et al., who reported transient hyphema as the most frequent postoperative complication in GATT procedures, with no long-term impact on outcomes [16]. Additionally, the conjunctival-sparing nature of GATT minimizes the risk of bleb-related complications, which are more commonly associated with traditional filtration surgeries.

The surgical success rates in this study were also favorable. Complete success, defined as achieving the target IOP without medications, was observed in 31.6% of the PHACO-GATT group and 26.9% of the standalone GATT group at 24 months (*p* = 0.876). Qualified success, defined as achieving the target IOP with or without medications, was achieved in 94.7% of the PHACO-GATT group and 96.2% of the standalone GATT group (*p* = 0.867). These results are comparable to the findings of Grover et al., who reported high rates of qualified success in GATT patients with various types of glaucoma [5]. The low rates of surgical failure in this study, with only 5.3% of the PHACO-GATT group and 3.8% of the standalone GATT group requiring additional glaucoma surgery, further underscore the durability of these procedures. Together, these findings reinforce the safety and efficacy of GATT, both as a standalone procedure and when combined with cataract surgery, in achieving long-term IOP control with minimal complications.

One of the key strengths of this study is the direct comparison of PHACO-GATT and standalone GATT over a two-year follow-up period, addressing an important gap in the current literature. While both procedures have been extensively studied independently, comparative data evaluating their relative efficacy and safety are limited. This study provides valuable insights into their outcomes in a real-world clinical setting, offering guidance for surgical decision-making in patients with moderate-to-severe OAG. Additionally, the use of standardized surgical protocols, consistent postoperative care, and predefined success criteria enhances the reliability and reproducibility of the findings. Another strength lies in the comprehensive analysis of multiple outcome measures, including IOP reduction, medication use, BCVA improvement, and RNFL stability. This multifaceted approach ensures that the results are clinically meaningful and reflect the holistic benefits of these procedures. Furthermore, the inclusion of patients with both POAG and PEG broadens the applicability of the findings, as these subtypes often present unique challenges in glaucoma management.

However, this study is not without limitations. The retrospective nature of the analysis introduces potential biases, such as selection bias and incomplete data recording. Additionally, the relatively small sample size, particularly when stratifying patients into the PHACO-GATT and standalone GATT groups, may limit the statistical power to detect subtle differences between the two procedures. Larger, multicenter studies are warranted to confirm these findings and provide more generalizable conclusions. Another notable limitation is the lack of long-term data beyond the 24-month follow-up period. Glaucoma is a chronic condition requiring lifelong management, and long-term studies are necessary to evaluate the durability of the observed outcomes, particularly in terms of structural stability and surgical success. Future research should also explore patient-reported outcomes, such as quality of life and satisfaction, to provide a more comprehensive assessment of these surgical approaches.

## 5. Conclusions

This study demonstrates that both PHACO-GATT and standalone GATT are effective and safe surgical options for managing moderate-to-severe OAG. Both procedures achieved significant and comparable reductions in IOP and medication use over a two-year follow-up period, with minimal complications and stable structural outcomes. PHACO-GATT offers the added benefit of cataract management, making it a particularly suitable option for patients with coexisting cataracts and glaucoma. These findings highlight the versatility and clinical utility of GATT as a MIGS, either as a standalone procedure or in combination with phacoemulsification. Further prospective, long-term studies are warranted to confirm these results and explore the durability of these outcomes in diverse patient populations.

## Figures and Tables

**Table 1:** Baseline patient demographic and ocular characteristics.

Characteristics	Group 1 (*n*:38)	Group 2 (*n*:26)	*p* Value
Mean age ± SD, years	74.4 ± 7.2	70.8 ± 7.3	0.061 ^a^
Gender, *n* (%)			0.935 ^b^
Female	15 (39.5)	10 (38.5)	
Male	23 (60.5)	16 (61.5)	
Glaucoma types, *n* (%)			0.911 ^b^
POAG	21 (55.3)	14 (53.8)	
PEG	17 (44.7)	12 (46.2)	
BCVA, logMAR ± SD (Median, IQR 25–75)	0.77 ± 0.29 (0.7, 0.51–1)	0.46 ± 0.19 (0.5, 0.3–0.51)	<0.001 ^c^
IOP ± SD mmHG (Median, IQR 25–75)	28.6 ± 6.3	27.04 ± 4.8 (28, 22.7–30)	0.365 ^c^
Number of AG medications ± SD (Median, IQR 25–75)	3.4 ± 0.8 (4, 3–4)	3.7 ± 0.5 (4, 3–4)	0.182 ^c^
Mean C/D rate ± SD (Median, IQR 25–75)	0.71 ± 0.19 (0.8, 0.6–0.8)	0.7 ± 0.15 (0.7, 0.6–0.8)	0.556 ^c^
Mean RNFL ± SD	67.8 ± 15.1	73.6 ± 11.2	0.099 ^a^
Mean CCT ± SD	531.9 ± 39.9	523.1 ± 25.2	0.326 ^a^

SD—standard deviation; BCVA—best-corrected visual acuity; IOP—intraocular pressure; C/D—cup/disc; AG—anti-glaucomatous; POAG—primary open-angle glaucoma; PEG—pseudoexfoliative glaucoma; RNFL—retinal nerve fiber layer; CCT—central corneal thickness. Mean ± standard deviation results are given in table (additionally, median and 25th and 75th percentiles are given in parentheses for nonparametric test results). *p* < 0,05 was considered statistically significant with 95% confidence interval (comparisons between Group 1 and Group 2). ^a^ Independent-samples *t*-test. ^b^ Chi^2^ test. ^c^ Mann–Whitney U test.

**Table 2:** Changes in intraocular pressure within groups separately during the study.

	Group 1 (*n*: 38)				Group 2 (*n*: 26)			
IOP, mm HG	Mean ± SD (Median, IQR 25–75)	95% CI of Mean, Lower/Upper	*p* Value	%95 CI of the Difference, Lower/Upper	Mean ± SD (Median, IQR 25–75)	95% CI of Mean, Lower/Upper	*p* Value	%95 CI of Difference, Lower/Upper
Preoperative	28.6 ± 6.3	26.6/30.7	<0.001 ^a^*		27 ± 4.8 (28, 22.8–30)	25.1/28.9	<0.001 ^a^*	
1st day	16.3 ± 4.5	14.8/17.8	<0.001 ^c^*	9.8/14.9	16.4 ± 6 (15, 14–18)	13.9/18.8	<0.001 ^b^*	8.2/13.1
1st month	14.2 ± 3.9	12.9/15.5	<0.001 ^c^*	12.4/16.5	14 ± 3.5 (13, 12.8–15)	12.6/15.4	<0.001 ^b^*	10.6/15.4
3rd month	13.8 ± 3.6 (14, 11–16)	12.6/14.9	<0.001 ^b^*	12.5/17.3	13 ± 2	12.2/13.9	<0.001 ^b^*	11.8/16.2
6th month	13 ± 2.8	12.1/13.9	<0.001 ^c^*	13.3/17.9	13.1 ± 2.3 (14, 12–14)	12.2/14	<0.001 ^b^*	11.6/16.2
12th month	12.8 ± 2.5	11.9/13.6	<0.001 ^c^*	13.5/18.1	12.9 ± 1.6	12.3/13.6	<0.001 ^b^*	11.9/16.3
24th month	12.7 ± 2.4 (13, 11–15)	11.9/13.5	<0.001 ^b^*	13.7/18.1	13 ± 1.7 (12, 12–14)	12.3/13.6	<0.001 ^b^*	11.8/16.2

SD—standard deviation; IOP—intraocular pressure; CI—confidence interval. Mean ± standard deviation results are given in table (additionally, median and 25th and 75th percentiles are given in parentheses for nonparametric test results). * *p* < 0.05 was considered statistically significant with 95% confidence interval (comparisons between baseline and other visits). ^a^ Friedman test. ^b^ Wilcoxon signed-rank test. ^c^ Paired sample *t*-test.

**Table 3:** Comparison of intraocular pressure (IOP) changes over time between Group 1 and Group 2.

	Group 1 (*n*: 38)		Group 2 (*n*: 26)		*p* Value	
IOP Changes (mmHG)	Mean ± SD	95% CI of Mean, Lower/Upper	Mean ± SD	95% CI of Mean, Lower/Upper		%95 CI of Difference, Lower/Upper
1st month	14.5 ± 6.2	12.4/16.5	13 ± 5.9 (14, 9.8–17.3)	10.6/15.4	0.306 ^a^	−1.6/4.6
3rd month	14.9 ± 7.3	12.4/17.3	14 ± 5.5 (15, 11.3–17.8)	11.8/16.2	0.747 ^a^	−2.5/4.3
6th month	15.6 ± 7.2	13.3/17.9	13.9 ± 5.7 (15, 10.8–17.3)	11.6/16.2	0.307 ^a^	−1.7/5
12th month	15.8 ± 6.9	13.5/18.1	14.1 ± 5.5 (16, 10–18)	11.9/16.3	0.287 ^a^	−1.5/5
24th month	15.9 ± 6.7	13.7/18.1	14.1 ± 5.4 (16, 10–18)	11.9/16.3	0.259 ^a^	−1.3/5

SD—standard deviation; CI—confidence interval; IOP—intraocular pressure. Mean ± standard deviation results are given in table (additionally, median and 25th and 75th percentiles are given in parentheses for nonparametric test results). *p* < 0.05 was considered statistically significant with 95% confidence interval. ^a^ Mann–Whitney U test.

**Table 4:** Comparison of mean number of anti-glaucomatous medications over time between Group 1 and Group 2.

	Group 1 (*n*: 38)			Group 2 (*n*: 26)			*p* Value	
Number of AG Medications	Mean ± SD (Median, IQR 25–75)	95% CI of Mean, Lower/Upper	*p* Value	Mean ± SD (Median, IQR 25–75)	95% CI of Mean, Lower/Upper	*p* Value		%95 CI of Difference, Lower/Upper
Preoperative	3.4 ± 0.8 (4, 3–4)	3.1/3.6	<0.001 ^b^*	3.6 ± 0.5 (4, 3–4)	3.4/3.8	<0.001 ^b^*	0.182 ^a^	−0.63/0.06
1st month	1.6 ± 1.2 (2, 0.8–2.3)	1.2/2	<0.001 ^c^*	1.9 ± 1.1 (2, 1–3)	1.5/2.4	<0.001 ^c^*	0.279 ^a^	−0.9/0.25
3rd month	1.5 ± 1.2 (2, 0–2)	1.1/1.8	<0.001 ^c^*	1.6 ± 1.1 (2, 0.5–2)	1.1/2	<0.001 ^c^*	0.548 ^a^	−0.7/0.45
6th month	1.2 ± 0.9 (1, 0–2)	0.9/1.5	<0.001 ^c^*	1.48 ± 0.8 (2, 1–2)	1.2/1.8	<0.001 ^c^*	0.116 ^a^	−0.77/0.14
12th month	1.1 ± 0.9 (1, 0–2)	0.8/1.5	<0.001 ^c^*	1.1 ± 0.9 (1, 0.5–2)	0.7/1.4	<0.001 ^c^*	0.965 ^a^	−0.43/0.5
24th month	1.2 ± 1 (1, 0–2)	0.9/1.6	<0.001 ^c^*	1.1 ± 0.8 (1, 0–2)	0.8/1.5	<0.001 ^c^*	0.785 ^a^	−0.38/0.57

SD—standard deviation; AG—anti-glaucomatous; CI—confidence interval. Mean ± standard deviation results are given in table (additionally, median and 25th and 75th percentiles are given in parentheses for nonparametric test results). * *p* < 0,05 was considered statistically significant with 95% confidence interval (comparisons between Group 1 and Group 2). ^a^ Mann–Whitney U test. ^b^ Friedman test. ^c^ Wilcoxon signed-rank test; comparison between preoperative value and present visit value.

**Table 5:** Comparison of success, complication, and failure rates.

	Complete Success	Qualified Success	Any Complications	Failure
At first year, *n* (%)				
Group 1 (*n*: 38)	13 (34.2)	38 (100)	26 (68.4)	N/A
Group 2 (*n*: 26)	10 (38.5)	26 (100)	18 (69.2)	N/A
*p*	0.728 ^a^		0.899 ^a^	
At second year, *n* (%)				
Group 1 (*n*: 38)	12 (31.6)	36 (94.7)	N/A	2 (5.3)
Group 2 (*n*: 26)	7 (26.9)	25 (96.2)	N/A	1 (3.8)
*p*	0.876 ^a^			0.867 ^a^

N/A—Not applicable. Complete success/qualified success: IOP decrease ≥20% from baseline or ≤21 mm Hg without medication or with a maximum of 2 medications. Failure: need for additional IOP-lowering surgery. *p* < 0.05 was considered statistically significant with 95% confidence interval (comparisons between Group 1 and Group 2). ^a^ Chi^2^ test.

## Data Availability

The data presented in this study are available on request from the corresponding author. The data are not publicly available due to ethical restrictions.
